# Identification of new polymorphic regions and differentiation of cultivated olives (*Olea europaea *L.) through plastome sequence comparison

**DOI:** 10.1186/1471-2229-10-211

**Published:** 2010-09-24

**Authors:** Roberto Mariotti, Nicolò GM Cultrera, Concepcion Muñoz Díez, Luciana Baldoni, Andrea Rubini

**Affiliations:** 1 CNR - Institute of Plant Genetics, Via Madonna Alta, 130, 06128 Perugia, Italy; 2 University of Cordoba - Dep. of Agronomy, Campus Universitario de Rabanales, 14080 Cordoba, Spain

## Abstract

**Background:**

The cultivated olive (*Olea europaea *L.) is the most agriculturally important species of the Oleaceae family. Although many studies have been performed on plastid polymorphisms to evaluate taxonomy, phylogeny and phylogeography of *Olea *subspecies, only few polymorphic regions discriminating among the agronomically and economically important olive cultivars have been identified. The objective of this study was to sequence the entire plastome of olive and analyze many potential polymorphic regions to develop new inter-cultivar genetic markers.

**Results:**

The complete plastid genome of the olive cultivar Frantoio was determined by direct sequence analysis using universal and novel PCR primers designed to amplify all overlapping regions. The chloroplast genome of the olive has an organisation and gene order that is conserved among numerous Angiosperm species and do not contain any of the inversions, gene duplications, insertions, inverted repeat expansions and gene/intron losses that have been found in the chloroplast genomes of the genera *Jasminum *and *Menodora*, from the same family as *Olea*.

The annotated sequence was used to evaluate the content of coding genes, the extent, and distribution of repeated and long dispersed sequences and the nucleotide composition pattern. These analyses provided essential information for structural, functional and comparative genomic studies in olive plastids. Furthermore, the alignment of the olive plastome sequence to those of other varieties and species identified 30 new organellar polymorphisms within the cultivated olive.

**Conclusions:**

In addition to identifying mutations that may play a functional role in modifying the metabolism and adaptation of olive cultivars, the new chloroplast markers represent a valuable tool to assess the level of olive intercultivar plastome variation for use in population genetic analysis, phylogenesis, cultivar characterisation and DNA food tracking.

## Background

Olive is the main cultivated species belonging to the monophyletic Oleaceae family, within the clade of Asterids, in which the majority of nuclear and organellar genomic sequences are unknown. The *Olea *genus includes two sections, *Olea *and *Ligustroides*. The former comprises the six recognised subspecies of the olive complex, which can be found throughout the Mediterranean area as well as the temperate and subtropical regions of Africa and Asia. The Mediterranean form (*Olea europaea*, subspecies *europaea*) includes the wild (var. *sylvestris*) and cultivated (var. *europaea*) olives [1].

Recently, chloroplast genome sequencing of species belonging to this family from the tribe of Jasmineae revealed that two genera, *Jasminum *and *Menodora*, carry several distinctive rearrangements, including inversions, gene duplications, insertions, inverted repeat expansions and gene/intron losses [2]. One of these genomic features involves the duplication of the *rpl*23 protein-coding gene in *Jasminum*. A similar duplication has also been detected in the Poaceae, and in both Oleaceae and Poaceae, the duplicated copy has been inserted into the intergenic region between *rbc*L and *psa*I [3]. By comparative gene mapping and sequencing, Lee and co-workers also demonstrated that all other Oleaceae genera, including *Olea*, have an identical gene content and order as *Nicotiana tabacum*. A phylogenetic reconstruction of the entire family, based upon the sequences of the *ndh*F and *rbc*L genes, partially confirmed previous results obtained by the analysis of the *trn*L-F and *rps*16 chloroplast regions [4].

Intraspecies variation within other Oleaceae genera, such as *Syringa *[5], *Forsythia *[6], *Ligustrum *[7] and *Fraxinus *[8,9] has also been examined.

Different chlorotypes have been identified among the six subspecies of *O. europaea*. Lumaret et al. [10] identified 12 distinct chlorotypes by RFLP analysis of DNA isolated from the purified chloroplasts of a wide set of *O. europaea *taxa. In other *O. europaea *subspecies Baldoni et al. [11] identified nine nucleotide substitutions, one insertion-deletion (indel) and a polymorphic poly-T SSR in the *trn*T-L region. Besnard et al. [12] in the *O. europaea *complex identified fourteen polymorphisms in three chloroplast regions (*trn*T-L, *trn*Q-R and *mat*K), including five microsatellite motifs, two indels and eight nucleotide substitution sites. Recently, the analysis of four regions (*trn*L-F, *trn*T-L, *trn*S-G and *mat*K) was used to demonstrate the polyphyletic origin of the *Olea *genus and estimate the divergence times for the major groups of *Olea *species and subspecies during the Tertiary period [13].

In cultivated olives chloroplasts are maternally inherited [14] and, in contrast to that seen at the subspecies level, a low plastidial variability was detected. A strong linkage disequilibrium between the chloroplast and mitochondrial genomes has been demonstrated, particularly for the Mediterranean cultivated and wild olives (subspecies *europaea*), suggesting that a low level of recurrent mutations occurs in both organellar genomes of the olive [15].

In particular, RFLP analysis of chloroplast DNA isolated from 72 cultivars revealed that most cultivars have a common chlorotype [16]. Besnard et al. [17], using two microsatellites and 13 RFLPs on more than 140 olive cultivars, were able to distinguish only four chlorotypes. The majority of cultivars was characterised by the chlorotype CE1, which likely originated from the wild olive populations of the Eastern Mediterranean and was spread to the Western part through cultivar dispersal by humans. Polymorphisms at the varietal level have been detected in the *trnD-T *locus [18], but only one polymorphism in this locus was found within a set of 12 cultivars [19].

Chloroplast DNA represents an ideal system for plant species DNA barcoding, and some chloroplast regions have been indicated as ideal for use in tests that discriminate between different land plants. Based on assessments of recoverability, sequence quality and discriminatory abilities at the species level, the two-locus combination of *rbc*L-*mat*K has been recommended as a universal framework for plant barcoding [20]. The combination of *trn*H-*psb*A coupled with *rbc*L has been recommended for DNA barcoding to discriminate between lower taxonomic ranks such as genera or related species [21]. In highly valuable crop species, such as the olive, that have a variety of cultivars available in the market, however, typing at the species level is not sufficient. Thus, the development of reliable methods to rapidly and efficiently discriminate between cultivars has become a pressing need. In addition, DNA barcoding may have useful applications to tracking food products [22] and the analysis of archaeological remains [23].

In this respect, the availability of complete chloroplast genome sequences from a growing number of species offers the opportunity to evaluate many potentially polymorphic sites and identify new regions that could be used to define cultivar DNA barcodes.

There are numerous approaches to sequence chloroplast genomes: traditional sequence analysis of highly purified chloroplast DNA, as applied for *Solanum lycopersicum *[24], *Lolium perenne *[25], *Trachelium caeruleum *[26], *Jasminum nudiflorum *[2] and *Parthenium argentatum *[27]; Rolling Circle Amplification (RCA) of high-purity chloroplast DNA, as demonstrated in *Cicer arietinum *[28], *Platanus occidentalis *[29] and *Welwitschia mirabilis *[30]; shot gun sequence analysis of BAC clones containing chloroplast genomic inserts, as demonstrated in *Vitis vinifera *[31], *Hordeum vulgare *[32] and *Brachypodium distachyon *[33]; and the use of universal primers based on chloroplast sequences highly conserved among most Angiosperm species to amplify overlapping fragments [34-36], as demonstrated in *Cycas taitungensis *[37] and two *Bambusa *species [38]. For this study, the last approach was used to sequence the entire chloroplast genome of the *O. europaea *subsp. *europaea *cv. Frantoio. The resulting availability of the entire plastome allowed to evaluate the sequence arrangement of the plastid genome in *O. europaea *and to identify new organellar polymorphisms that could discriminate between cultivated olive varieties.

## Results and Discussion

### Size, gene content and gene order of the olive chloroplast genome

**Figure 1:**
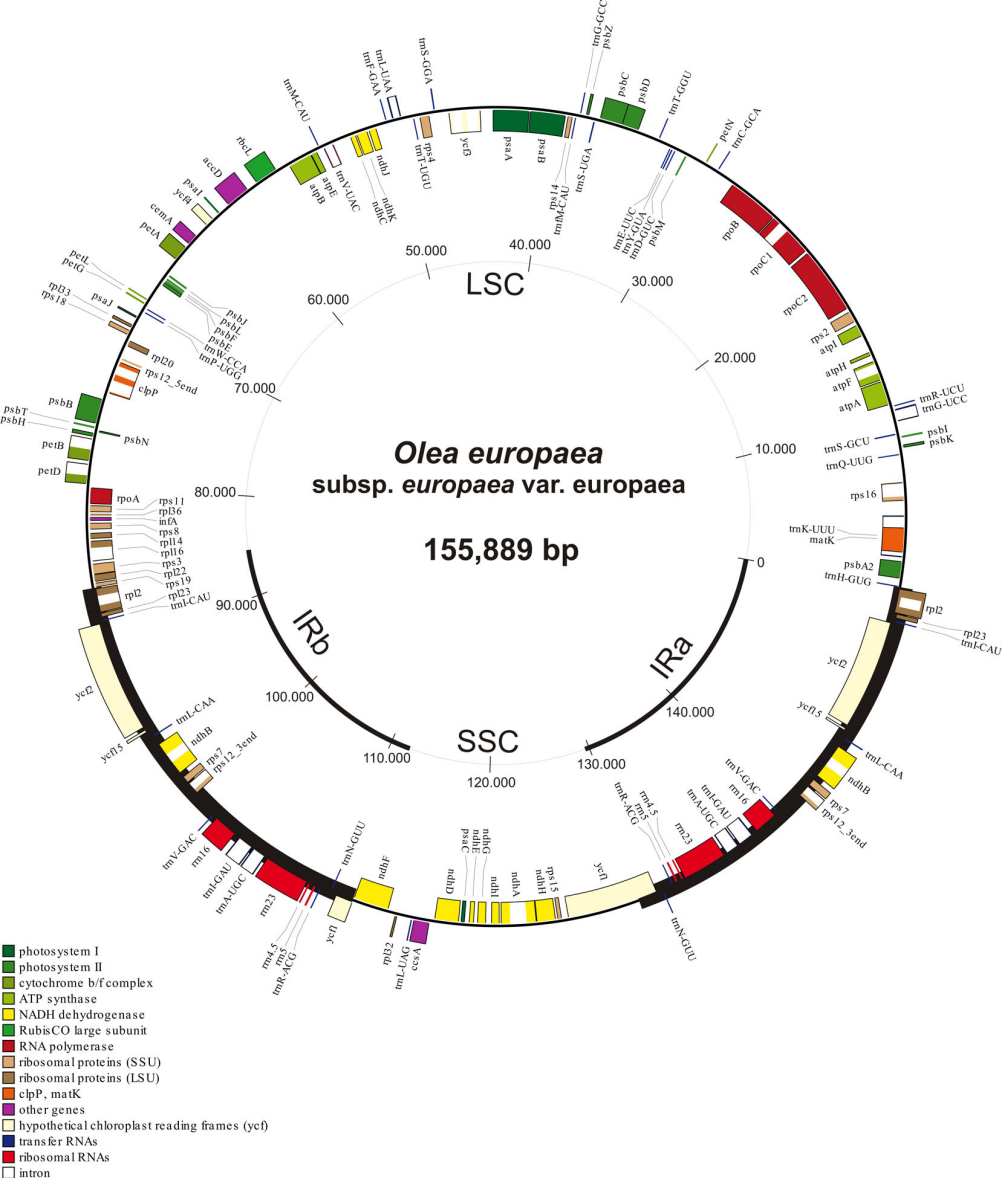
***Olea europaea *chloroplast genome based on direct sequencing of the LSC, SSC and IR regions**. Genes drawn inside the circle are transcribed clockwise, those outside are counterclockwise.

The complete plastome of olive, cv. Frantoio has a total length of 155,889 bp (GenBank Accession Number GU931818), with the typical structure found in the unrearranged chloroplast genomes of Angiosperms. It includes an 86,614-bp Large Single Copy (LSC) and a 17,791-bp Small Single Copy (SSC) region separated by a pair of Inverted Repeats (IR), each 25,742 bp long (Figure 1). Coding DNA (92,095 bp) accounts for 59.08% of the genome and includes protein coding genes (80,252 bp), tRNAs (2,793) and rRNAs (9,050), while noncoding DNA (63,794 bp) accounts for the remaining 40.92% and includes introns (20,130 bp) and intergenic spacers (43,664 bp). The olive plastome contains 114 unique genes (80 CDS, 30 tRNA and 4 rRNA), with 19 of these genes (8 CDS, 7 tRNA and all 4 rRNA) duplicated in the IR for a total of 133 genes. In addition, the duplicated region includes a partial CDS for *ycf*1, as in other species like *Typha *[39]. There are 18 intron-containing genes, 15 of which contain one intron and 3 (*ycf*3, *clp*P and *rps*12) with two introns. The *rps*12 gene is trans-spliced, with the 5' end located in the LSC and the 3' end duplicated in the IR regions. The nucleotide composition of the olive chloroplast genome comprises 37.81% GC and 62.19% AT.

The *in silico *search for repetitive elements identified 633 mono-nucleotide SSRs with 5 or more repeat units (Table 1), with 276 poly-A, 303 poly-T, 31 poly-C and 23 poly-G repeats. In addition, six di-nucleotide SSRs with five or six repeat units, no tri-nucleotide SSRs, three tetra- and two penta-nucleotide SSRs were identified, for a total of 644 repetitive sequences. The distribution of SSRs across the chloroplast genome was as follows: 400 in the LSC (density = 0.0046), 126 in the SSC (density = 0.0071) and 59 (x2) in the IR region (density = 0.0022).

**Table 1:** Abundance and length of SSR motifs identified on the olive chloroplast genome.

No. of repeats*	No. of SSR												
	**A**	**C**	**G**	**T**	**AT**	**GA**	**TA**	**AAAT**	**ATAA**	**GAAA**	**CCAAT**	**TAAAC**	**Total**

3	-	-	-	-	0	0	0	0	**1**	**1**	**1**	**1**	4
4	-	-	-	-	0	0	0	**1**	0	0	0	0	1
5	-	-	-	-	**1**	**1**	**3**	0	0	0	0	0	5
6	141	20	17	161	0	**1**	0	0	0	0	0	0	340
7	69	8	6	57	0	0	0	0	0	0	0	0	140
8	23	2	0	33	0	0	0	0	0	0	0	0	58
9	20	0	0	19	0	0	0	0	0	0	0	0	39
10	**11**	**1**	0	**14**	0	0	0	0	0	0	0	0	26
11	**4**	0	0	**9**	0	0	0	0	0	0	0	0	13
12	**5**	0	0	**7**	0	0	0	0	0	0	0	0	12
13	**1**	0	0	**2**	0	0	0	0	0	0	0	0	3
14	**1**	0	0	0	0	0	0	0	0	0	0	0	1
15	**1**	0	0	0	0	0	0	0	0	0	0	0	1
16	0	0	0	**1**	0	0	0	0	0	0	0	0	1
Total	276	31	23	303	1	2	3	1	1	1	1	1	644

SSRs analyzed for polymorphism are given in bold. * Mononucleotide SSRs with less than 6 repeats were not determined.

Interspersed repeats and palindromic sequences.

**Figure 2:**
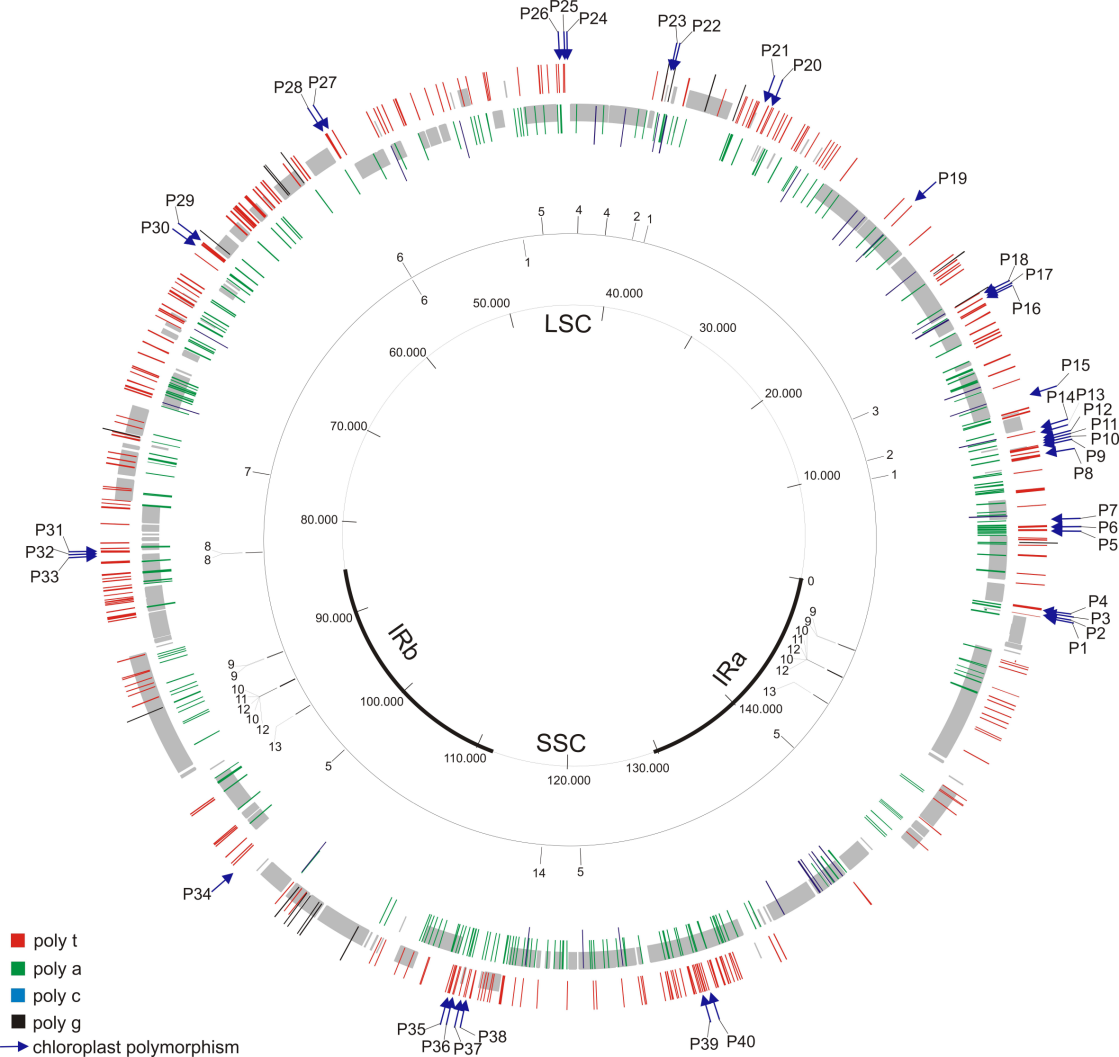
**Polymorphic regions identified in the olive chloroplast genome**. Different colours indicate the four mono-nucleotide microsatellites (poly-T and poly-G are reported in the external circle, poly-A and poly-C in the internal circle), bar lengths correspond to the number of repetitions. Arrows indicate polymorphisms (base mutations, microsatellites and indels). The circle reports the interspersed repeats: to the same number corresponds the same repetition. External or internal number position corresponds to the sense or anti-sense sequence direction.

The repeat analysis also identified 14 interspersed repetitive sequences longer than 30 bp, each having 2-6 repetitions and a sequence identity higher than 85% (Table 2, Figure 2). These long interspersed repetitive sequences included two tandem repeats in the *ycf*2 gene and five palindromic sequences (two in the LSC, one in the SSC and two in the IR regions). Three of the four repeats found within the *ycf*2 exon were tandem repeats, as previously observed in *V. vinifera *[31]. There were only two inverted repeats, all the others were direct repeats. Five repeats were located within CDS, two repeats were found in the introns of the *ycf*3 and *ndh*A genes and all others were in the intergenic spacers (Table 2). Interspersed repeats did not cause any uncertainty during the sequencing process because they were quite short (< 61 bp), with a low number of repetitions and primers were never constructed on the repeats.

**Table 2:** 

Repeat	Number of repeats	Size	**Start**^(1)^	Type	% Identity	Region	Gene position	Sequence
1	3	30	9,345	D	86.67	LSC	*trn*S-GCU-exon	[CA][AC]GGA[GA]AGAGAGGGATTCGAACCCTCG[AG]TA
			37,281	D		LSC	*trn*S-UGA-exon	
			47,117	I		LSC	*trn*S-GGA-exon	
2	2	31	10,849	D	90.32	LSC	*trn*G-UCC-exon 2	[AT][AG]A[CA]GATGCGGGTTCGATTCCCGCTA[CT]CCGC
			38,241	D		LSC	*trn*G-GCC-exon	
3	1	30	14,401	P	93.33	LSC	*atp*F - *atp*H	AAATATGAAAAATA[TC][GA]TATTTTTCATATTT
4	2	45	40,451	D	88.89	LSC	*psa*B-exon	[AT]TGCAATAGCTA[AG]ATGATG[AG]TG[TA]GCAATATCGGTCAGCCATA[AG]AC
			42,675	D		LSC	*psa*A-exon	
5	3	41	45,474	D	92.68	LSC	*ycf*3-intron	T[CA]CAGAACCGTAC[GA]TGAGATTTTCA[TC]CTCATACGGCTCCTC
			100,797	D		IR	*rps*12 - *trn*V-GAC	
			122,052	D		SSC	*ndh*A-intron	
6	2	31	56,736	D	90.32	LSC	*atp*B - *rbc*L	T[AT]CTTATTCATCCACTTGAAATTTTCAA[AG][AT]T
			56,777	I		LSC	*atp*B - *rbc*L	
7	1	44	76,926	P	95.45	LSC	*psb*T - *psb*N	TTGAAGTAATGAGCCTCCC[CA]ATAT[TG]GGGAGGCTCATTACTTCAA
8	2	30	83,181	D	90	LSC	*rps*8 - *rpl*14	AATCTA[CG]T[AT][AC]**TTAATCTAGTTC**TTAATCTA
			83,193	D		LSC	*rps*8 - *rpl*14	
9	2	30	91,385	TR	90	IR	*ycf*2-exon	TTTCTTTTTGTC[CT]AA[GC]TCACTTC[TC]TTTTTT
			91,427			IR	*ycf*2-exon	
10	2	36	93,791	TR	94.83	IR	*ycf*2-exon	[AG]ATATTGATG[AC]TAGTGAC[AG]ATATTGATG[AC]TAGTGAC
			93,827			IR	*ycf*2-exon	
11	1	48	96,252	P	91.67	IR	*ycf*15 - *trn*L-CAA	AGAGCTCGGATCGAATCGGTAT[TA][TG][AC][TA]ATACCGATTCGATCCGAGCTCT
12	2	30	109,623	D	93.33	IR	*rrn *4.5 - *rrn *5	CATTGTTCAA[AC]TCTTTGACAACA[CT]GAAAAA
			109,654	D		IR	*rrn *4.5 - *rrn *5	
13	1	61	110,599	P	95.08	IR	*trn*R-ACG - *trn*N-GCU	AGAATTCTCAGATGTACTAGCACTGCATC[AT][AT][AT]GATGCAGTGCTAGTACATCTGAGAATTCT
14	1	36	118944	P	100	SSC	*ndh*D - *psa*C	AAAACCCGTGCTCCAAATATTTGGAGCACGGGTTTT

D: direct, I: inverted, P: palindrome, TR: tandem repeat (imperfect).

^(1) ^The start base position of the interspersed repeats and palindromic sequences refers to the cv. Frantoio sequence.

Bold nucleotides refer to the indel P32.

The actual size of the olive plastome is larger than the size estimated on the basis of RFLP analysis, which predicted a range from 132 to 134 Kb [16].

### Olive chloroplast genome organisation

The sequence of the olive chloroplast genome represents one of the first contributions to deciphering the genetic background of this important tree crop species and was used to verify that rearrangements observed in the plastomes of other genera of Oleaceae, such as *Jasminum *and *Menodora*, were not represented in *O. europaea*. In fact, in contrast to what observed in the *Jasminum *and *Menodora *plastomes [2], the olive chloroplast maintains a size range, organisation and gene order typical of most land plants, such as members of the *Vitis*, *Populus*, *Citrus*, *Eucalyptus*, *Coffea *and *Arabidopsis *genera. Based on the phylogeny of Oleaceae inferred from the *ndh*F and *rbc*L genes [2], *Jasminum *and *Menodora *were already known to be unusual genera within the family, and all other tribes, including Oleae, to which the *Olea *genus belongs, do not share their combination of multiple mutational events. The highly conserved plastome organization of the olive allowed universal primers and genome walking with consensus primers to be used to amplify most of the LSC region.

### Identification of new plastid markers to discriminate between olive cultivars

To detect intervarietal polymorphisms, a preliminary screening of the intergenic spacer *trn*S-GCU - *trn*G-UCC, previously demonstrated to be polymorphic among olive varieties [40], was performed on a set of 30 cultivars having different geographical distributions and representing a wide range of morphological and agronomical phenotypes (data not shown). A sub-set of eight highly variable cultivars (Table 4) was further examined for 100 potentially polymorphic regions.

The tested potential variant domains have shown different levels of variability. Fifteen of the analyzed intergenic spacers contained mutations within the sequence of the eight cultivars, ranging in number from one to six per region. These mutations were microsatellites, indels or single nucleotide polymorphisms (Table 3). One SNP was located within the intron of the *rpo*C1 gene, and three others were located in the coding regions (CDS) of the *rpl*14, *ndh*F and *ycf*1 genes. The CDS-SNPs resulted in substitutions at aminoacidic position 109 in *rpl*14 (leucine to phenylalanine), at 32 aa in *ndh*F (valine to alanine), and at 995 and 1,161 aa in *ycf*1 (leucine to isoleucine and isoleucine to arginine, respectively). Blast analyses revealed that the *ndh*F alanine and the *ycf*1 leucine, widely represented in other species, are present in Farga and Frantoio cultivars, respectively. Also the *rpl*14 polymorphism can be found in other species, as is the case for the phenylalanine aminoacid, present in the *V. vinifera *cv. Pinot Noir in the mitochondrial copy of this gene, due to the incorporation of more than 42% of the *Vitis *chloroplast genome into its mitochondrial genome [41]. On this respect, the risk that our chloroplast olive markers may reside on mitochondrial or nuclear genes has been prevented by amplifying coding regions anchored on the intergenic spacers and confirmed by the absence of sequence ambiguities.

**Table 3:** Chloroplast polymorphisms within olive (*Olea europaea *subsp. *europaea *var. europaea) cultivars.

Polymorphic sites	Marker	Polymorphism type	Motif	Position (bp)^(1)^	Region	Polymorphisms already known (Authors)
**P1**	*Oe-rpl2-trn*H	SNP	A/G	2	*rpl2-trn*H	
**P2**	*Oe-trn*H-*psb*A-1	SNP	A/T	221		
**P3**	*Oe-trn*H-*psb*A-2	SNP	C/T	470	*trn*H-GUC - *psb*A2	
**P4**	*Oe-trn*H-*psb*A-3	SSR	T_10-12_	505		
**P5**	*Oe-trn*K-*rps*16-1	SSR	T_11-12_	4,690	*trn*K-UUU - *rps*16	
**P6**	*Oe-trn*K-*rps*16-2	SSR	T_11-12_	4,883		
**P7**	*Oe-trn*K-*rps*16-3	SNP	A/C	5,011		
P8	*Oe-psb*K-*psb*I	SSR	T_10-11_	9,072	*psb*K - *psb*I	Besnard et al., 2003
**P9**	*Oe-trn*S-*trn*G-1	SNP	C/T	9,463	*trn*S-GCU - *trn*G-UCC	
**P10**	*Oe-trn*S-*trn*G-2	SNP/indel	A/T/-	9,535		
P11	*Oe-trn*S-*trn*G-3	Indel	TTAGATA/-	9,536		Besnard et al., 2003
P12	*Oe-trn*S-*trn*G-4	Indel	A_4_(G)A_5_/-	9,574		Besnard et al., 2003
P13	*Oe-trn*S-*trn*G-5	SSR	A_11-14_	9,579		Besnard et al., 2003
**P14**	*Oe-trn*S-*trn*G-6	SNP	G/T	9,960		
**P15**	*Oe-atp*A-*atp*F	SSR	A_15-16_	12,790	*atp*A - *atp*F	
P16	*Oe-rps*2 - *rpo*C2-1	SSR	C_10-11_	17,433	*rps*2 - *rpo*C2	Besnard et al., 2007 (*ccmp5*)
P17	*Oe-rps*2 - *rpo*C2-2	SSR	T_10-11_	17,443		Besnard et al., 2007 (*ccmp5*)
P18	*Oe-rps*2 - *rpo*C2-3	SSR	A_12-13_	17,455		Besnard et al., 2007 (*ccmp5*)
**P19**	*Oe-rpo*C1	SNP	C/T	23,981	*rpo*C1 intron	
P20	*Oe-trn*E-*trn*T-1	SSR	A_12-13_	32,682	*trn*E-UUC - *trn*T-GGU	Intrieri et al., 2007; Besnard, 2008
P21	*Oe-trn*E-*trn*T-2	SNP	C/T	32,813		Intrieri et al., 2007
**P22**	*Oe-psb*Z-*trn*G-1	SSR	A_10-11_	38,011	*psb*Z - *trn*G-GCC	
**P23**	*Oe-psb*Z-*trn*G-2	SNP	C/T	38,129		
**P24**	*Oe-psa*A-*ycf*3-1	SNP	A/G	43,868	*psa*A - *ycf*3	
**P25**	*Oe-psa*A-*ycf*3-2	SSR	A_11-12_	44,077		
**P26**	*Oe-psa*A-*ycf*3-3	SNP	C/T	44,302		
**P27**	*Oe-atp*B-*rbc*L-1	SNP	A/G	56,929	*atp*B - *rbc*L	
P28	*Oe-atp*B-*rbc*L-2	SSR	A_6-7_	57,116		Besnard et al., 2007 (*ccmp7*)
**P29**	*Oe-pet*A-*psb*J-1	SNP	C/T	65,656	*pet*A - *psb*J	
**P30**	*Oe-pet*A-*psb*J-2	SNP	G/T	66,340		
**P31**	*Oe-rps*8-*rpl*14-1	SSR	T_11-18_	83,112	*rps*8 - *rpl*14	
**P32**	*Oe-rps*8-*rpl*14-2	Indel	TTAATCTAGTTC/-	83,195		
**P33**	*Oe-rpl*14	SNP	G/T	83,307	*rpl*14 exon	
**P34**	*Oe-rps*12-*trn*V-1	SNP	A/T	101,265	*rps*12 - *trn*V-GAC	
**P35**	*Oe-ndh*F	SNP	A/G	114,454	*ndh*F exon	
**P36**	*Oe-ndh*F-*rpl*32	SSR	T_9-10_	114,885	*ndh*F - *rpl*32	
**P37**	*Oe-rpl*32-*trn*L-1	SSR	A_14_/A_7_(T)A_5_	115,359	*rpl*32 - *trn*L-UAG	
**P38**	*Oe-rpl*32-*trn*L-2	SNP	A/C	115,598		
**P39**	*Oe-ycf*1-1	SNP	A/C	127,793	*ycf*1 exon	
**P40**	*Oe-ycf*1-2	SNP	G/T	128,292		

The position of the polymorphic regions refers to the cv. Frantoio sequence.

The new markers identified on olive cultivars are given in bold.

**Table 4:** Chlorotypes detected on eight cultivars.

Repository of samples/Collection number	CRA-OLI/92	CRA-OLI^1^/32	WOGB^2^/12, 691	WOGB/128	WOGB/5, 787	WOGB/114
**Polymorphic sites**	**Chloroptype 1** **(Frantoio)**	**Chloroptype 2** **(Canino)**	**Chloroptype 3** **(Farga, Kalogerida)**	**Chloroptype 4** **(Galega)**	**Chloroptype 5** **(Lechin Sevilla, Sorani)**	**Chloroptype 6** **(Oueslati)**

P1	G	A	A	G	A	A
P2	T	A	T	T	T	A
P3	T	C	T	T	C	C
P4	T_12_	T_11_	T_10_	T_12_	T_11_	T_11_
P5	T_11_	T_12_	T_12_	T_11_	T_12_	T_12_
P6	T_12_	T_11_	T_12_	T_12_	T_11_	T_11_
P7	A	A	C	A	A	A
P8	T_11_	T_10_	T_11_	T_11_	T_10_	T_10_
P9	T	C	C	T	C	C
P10	A	/	A	A	T	/
P11	TTAGATA	-	TTAGATA	TTAGATA	-	-
P12	-	A_4_(G)A_5_	A_4_(G)A_5_	-	A_4_(G)A_5_	A_4_(G)A_5_
P13	A_12_	A_14_	A_11_	A_12_	A_14_	A_14_
P14	T	G	T	T	G	G
P15	A_15_	A_16_	A_16_	A_15_	A_16_	A_16_
P16	C_10_	C_11_	C_11_	C_11_	C_10_	C_10_
P17	T_11_	T_10_	T_10_	T_11_	T_10_	T_10_
P18	A_12_	A_12_	A_13_	A_12_	A_12_	A_12_
P19	C	T	C	C	T	T
P20	A_13_	A_13_	A_12_	A_13_	A_13_	A_13_
P21	C	C	T	C	T	C
P22	A_10_	A_10_	A_11_	A_10_	A_10_	A_10_
P23	C	C	T	C	C	C
P24	A	A	G	A	A	A
P25	A_12_	A_12_	A_11_	A_12_	A_12_	A_12_
P26	C	C	C	C	T	C
P27	A	G	G	A	G	G
P28	A_6_	A_7_	A_6_	A_6_	A_7_	A_7_
P29	C	T	C	C	C	T
P30	T	G	G	G	G	G
P31	T_18_	T_11_	T_18_	T_17_	T_11_	T_11_
P32	TTAATC TAGTTC	TTAATCTAGTTC	-	TTAATC TAGTTC	TTAATCTAGTTC	TTAATC TAGTTC
P33	T	G	G	G	T	T
P34	T	A	T	T	A	A
P35	A	G	G	A	G	G
P36	T_10_	T_9_	T_9_	T_10_	T_9_	T_9_
P37	A_14_	A_7_(T)A_5_	A_7_(T)A_5_	A_14_	A_7_(T)A_5_	A_7_(T)A_5_
P38	C	A	A	C	A	A
P39	A	A	C	A	A	A
P40	G	G	T	G	G	G

The position of the polymorphic regions refers to the cv. Frantoio sequence.

The comparison of the Frantoio chloroplast sequence with ESTs deriving from fruits of cvs. Coratina and Tendellone showed some sequence mismatches, but they were not confirmed by resequencing the corresponding genomic regions in Coratina and Tendellone cultivars.

Overall, the analysis of cpDNA sequences from the eight cultivars resulted in the identification of 40 polymorphic sites, 30 of which represent new and never-described plastid variants (Table 3, Table 4, Figure 2). Sixteen polymorphic sites were mono-nucleotide SSRs: eight poly-A, including one with an irregular motif; seven poly-T and one poly-C. The remaining polymorphisms included 20 SNPs and 4 indels. Thirty-three polymorphic sites (P1-P33) were located within the LSC region, one (P34) within the IR and six (P35-P40) within the SSC (Figure 2). The indel P32 was identified within the repeat of the *rps*8 - *rpl*14 spacer, but none of the other repetitive regions was polymorphic between cultivars.

The chloroplast sequence of cv. Frantoio was also compared with all previously sequenced regions of the olive chloroplast, particularly with the plastome sequence of cv. Bianchera, which has been recently deposited in the Genbank database (NC_013707.1). More than 200 mismatches were detected between the Bianchera and Frantoio sequences. Surprisingly, not one of these polymorphisms fell within the previously identified cultivar-specific polymorphic regions. To verify if these mismatches might represent real sequence differences between the two varieties, most of the ambiguous regions were reamplified and resequenced in both cultivars (Bianchera sample was provided by the CRA-OLI of Spoleto, Perugia, Italy). These analyses confirmed the sequence of cv. Frantoio and showed an absolute sequence identity with that obtained from the cv. Bianchera in all of these regions, including the exons of the *rpo*C1 and *ndh*F genes, carrying 27- and 9-bp indels, respectively. The differences detected between the two olive plastome sequences can not derive from an incorrect identification of the Bianchera genotype because, in that case, mutations should have been found in the polymorphic sites and not randomly along the chloroplast genome. More likely, divergences may be attributed to sequence uncertainties in the Bianchera plastome sequence deposited in GenBank.

The new markers identified in this study can distinguish six haplotypes among eight cultivars. Therefore, these new markers hold great promise for the identification of new cultivar haplotypes and for use in DNA barcoding systems to distinguish between different cultivars.

### Comparison of plastome variation between cultivars and with other *Olea *taxa

Based on previous chloroplast sequence analyses, olive cultivars belong to the cp-II lineage and have been classified into three sublineages (E1, E2 and E3) and four chlorotypes (1, 2, 9 and 13) [19,40]. These chlorotypes were defined by evaluating length variations in the *psb*K-*psb*I, *trn*S-*trn*G, *rps*2-*rpo*C2, *trn*E-*trn*T and *atp*B-*rbc*L regions among more than 140 cultivars [17,19,40].

Several polymorphisms had been previously identified in the partial sequence of the *trn*K intron (AF359497-AF359504) by analysing the subspecies *cuspidata, laperrinei*, *maroccana*, *cerasiformis*, *guanchica*, *europaea *var. sylvestris (wild olive) and the Cornicabra cultivar, but none of these polymorphisms were found among the cultivars we have analysed. The *psb*K-*psb*I and *trn*S-GCU-*trn*G-UCC regions, spanning the polymorphic sites P8, P9, P10, P11, P12, P13 and P14, were analyzed by Besnard et al. [12] as fragment length variation on a set of different *O. europaea *taxa including cultivars. That analysis revealed intercultivar variability only at P11, P12 and P13 but was unable to keep the C/T and G/T SNPs in the P9 and P14 sites, respectively. We treated the A/T/- polymorphism, closely linked to P11, as a different polymorphism (P10) because the A/- indel is present in most varieties while the T is a rare mutation carried by few cultivars.

The spacer *rps*2-*rpo*C2, spanning the polymorphic sites P16, P17 and P18, generated five different chlorotypes among the eight varieties analysed, demonstrating a high level of rearrangement within cultivars. This region corresponds to the *ccmp5 *microsatellite [42,43], but previous studies that analysed only length polymorphisms were unable to capture the complexity of this region. P28 includes *ccmp7 *[40,42] and an additional SNP polymorphism (P27) captured in the flanking region.

Intrieri et al. [18] reported the identification of 5 SNPs and 4 indels in the *trn*D-*trn*T region of 13 cultivars. Analyzing a different set of cultivars, Besnard [19] did not detect these polymorphisms. Similarly, only two polymorphisms were confirmed in our cultivar set: the poly-A SSR (P20) and the C/T SNP (P21).

Other regions previously analysed in different *Olea *taxa, such as *trn*L-F and *rps*16 [4], *trn*L-*trn*F [13], and *trn*T-*trn*L [11] were not polymorphic among our cultivars.

No differences between the eight cultivars were found within the *mat*K and *psb*A exons or the *rps*16 intron, regions used for species barcoding. In contrast, the *psb*K*-psb*I and *trn*H*-psb*A barcoding regions, both representing markers for plant species identification [44,45], correspond to our P8, P2, P3 and P4 polymorphisms. This observation indicates that these markers may not accurately discriminate between some species, given their potential intra-specific genetic variations [46].

## Conclusions

The low level of cpDNA variation detected up to now within olive cultivars represented a serious obstacle to the widespread use of cpDNA markers for cultivar characterization, parentage analysis and population genetics. The most probable causes of the high level of sequence conservation may be related to the domestication process, by which most cultivars were likely derived from only a few different wild plants, and the low generation turnover resulting from the long life span of the trees, which reduces the rate of emergence of new mutations.

In this study, using eight cultivars, 30 new cpDNA markers were identified from the olive plastome sequence and 10 markers previously reported were confirmed. In fact, the availability of the entire chloroplast genome and systematic sequencing of candidate regions from selected cultivars resulted in the identification of many new polymorphisms, mostly represented by nucleotide substitutions and by rearrangements of different microsatellites. They were not discovered in previous analyses likely because these focused mostly on fragment length variations.

The 40 markers applied to eight cultivars were able to split them into six different chlorotypes. The ten known markers are able to establish to which lineages the olive varieties may correspond and to reconstruct their phylogeny with potential ancestors, while the new markers should allow to break down cultivated olives into new chlorotypes and to finely assign them to different lineages within the Mediterranean *O. europaea *complex. These markers could provide a valuable contribution to understanding the evolutionary and ecological processes involved in olive domestication as well as to increase the knowledge about the function of plastid genes on plant metabolism.

They could be used to screen olive genotypes, to assess the chlorotype distribution among cultivars and to better determine their phylogenetic relationships with the wild populations as well as with other *O. europaea *subspecies. This could help reconstruct the origin of the cultivated olive and to determine the timeline involved in the distribution of chlorotypes from traditional varieties throughout the Mediterranean region.

Most of these polymorphisms showed a high level of reorganization among cultivars, particularly in the intergenic regions such as *psa*A-*ycf*3, *rps*2-*rpo*C2 and *trn*S-GCU-*trn*G-UCC. This observation demonstrates that after rearrangements occurred within the plastid genome, these changes were fixed and maintained within cultivars by vegetative propagation. The putative functional role that these mutations may play in modifying the metabolism of olive cultivars and in developing adaptations to the environment, will also represent a further contribution to understanding the genetic background of the olive, providing insights into the evolution of plant phenotypes. The application of these polymorphisms as functional markers will also be considered.

Finally, these polymorphisms represent a new source of markers for olive DNA barcoding to distinguish between cultivars, for practical applications related to DNA-based tracking of olive oil and the identification of archaeological remains. One particular focus involves their potential use in DNA tracking of food products derived from the olive (e.g., olive oil and table olives), based on the assumptions that: i) the high number of chloroplasts per cell increases the probability that trace amounts of DNA can be amplified from these food products; ii) their maternal origin excludes the risk that DNA from pollinators would be amplified instead; iii) the haploid chloroplast genome can produce cultivar-specific single signals.

The identification of 30 new polymorphic sites, most of which are located in chloroplast regions previously unexplored in cultivated *O. europaea*, demonstrates that chloroplast variation in olive cultivars is higher than expected and that new chlorotypes could be discovered through the analysis of a larger number of cultivars.

## Methods

### Plant material and DNA extraction

For the plastome sequence analysis, leaves of cv. Frantoio were collected from the accession present at the CRA-OLI olive cultivars collection (Collececco, Spoleto).

For the detection of intervarietal polymorphisms, a subset of eight cultivars was used, chosen among 30 cultivars pre-selected on the basis of their haplotypes for the intergenic *trn*S-GCU - *trn*G-UCC spacer (Table 4).

Total DNA was extracted by the DNeasy Plant Mini Kit (Qiagen, Hilden, Germany), following the manufacturer's instructions.

### Sequencing strategy: primer design and PCR amplification

Sequencing of the olive plastome was performed by designing a series of PCR primer pairs that produced partially overlapping amplicons and spanned the entire chloroplast genome.

For the Large Single Copy (LSC) region, 38 primer pairs located within conserved regions and designed by Grivet et al. [34] were used, avoiding gaps between successive fragments along the cpDNA molecule. Five primer pairs (5-14-22-27-38) produced double bands, and two (16-28) did not produce any amplification. Thus, new primers for those regions were constructed, following the strategy used for the amplification of the IR and SSC regions. For primer sequences see Additional File 1, Table S2.

For the SSC and the IR regions, primers were constructed from conserved sequences derived by the alignment of the plant chloroplast genomes of *Jasminum nudiflorum *(DQ673255), *Populus tricocharpa *(EF489041), *Vitis vinifera *(DQ424856), *Eucaliptus globulus *(AY780259), *Arabidopsis thaliana *(AP000423), *Gossypium hirsutum *(DQ345959), *Citrus sinensis *(DQ864733), *Cucumis sativus *(AJ970307), *Morus indica *(DQ226511), *Panax ginseng *(AY582139), *Solanum lycopersicum *(AM087200) and *Nicotiana tabacum *(Z00044). These sequences were retrieved from GenBank and aligned using Muscle V. 3.7 [47], and the primers were designed using PerlPrimer v1.1.6 [48]. Because the average size of the amplified fragments was approximately 2,500 bp, internal primers to sequence the entire amplicons were also designed. The primer sequences and positions, along with their respective amplicon lengths, are given in Additional File 1, Table S1.

PCR amplifications were performed in a final volume of 50 μL containing 1-20 ng of template DNA, 10× PCR buffer, 200 μM of each dNTP, 10 pmol of each primer and 2 U of EuroTaq polymerase (EuroClone). For those fragments that were longer than 5,000 bp, 1 unit of LA Taq polymerase (TaKaRa) was used instead. The amplifications were performed with the PCR System 9600 (Applied Biosystems, Foster City, CA) using the following cycling conditions: an initial denaturation step of 95°C for 5 min, followed by 35 cycles of 95°C for 30 sec, 60°C for 30 sec and 72°C for 25 sec and a final elongation step of 72°C for 30 min. For those amplifications including LA Taq polymerase in the PCR mix, the following cycling conditions were used instead: an initial denaturation step of 94°C for 1 min, followed by 30 cycles of 98°C for 60 s and 68°C for 10 min and a final extension step of 72°C for 10 min. Negative controls (no template DNA) were included in all experiments.

The PCR products were checked by electrophoresis on 2% agarose gels, then purified with the JetQuick PCR purification kit (Genomed) and directly sequenced in both directions using the ABI Prism BigDye Terminator V.3.1 Ready Reaction Cycle Sequencing Kit (Applied Biosystems) on an ABI 3130 Genetic Analyzer (Applied Biosystems-Hitachi). The sequences were assembled using BioEdit v7.0.9 software (Ibis Biosciences, Carlsbad, CA).

The DOGMA program [49] was used for the initial genome annotation, which was then manually refined using Artemis version 11 [50] and NCBI Blast searches. The annotation of tRNA genes was checked using tRNAscan version 1.21 [51]. The genome map was generated using OGDRAW software V. 1.0 [52].

### Evaluation of repeat structures

Msatfinder v. 2.0.9 [53] was used to identify simple sequence repeats (SSR), with the following settings: a six-repeat threshold for mono-nucleotide SSRs, a five-repeat threshold for di- and tri-nucleotide SSRs and a three-repeat threshold for tetra-, penta- and esa-nucleotide SSRs. The SSR density in the different regions of the chloroplast genome was calculated by dividing the number of SSRs by the length of the given region. Interspersed repeats were identified with REPuter [54] by setting the minimum repeat size to 30 bp and the Hamming distance to 3. The presence and distribution of the repetitive element were verified manually using Artemis and computationally by performing an intragenomic Blast search. For this purpose, the sequence was interrogated using a local installation of NCBI Blast and a Blast database created with formatDB software http://www.ncbi.nlm.nih.gov/staff/tao/URLAPI/wwwblast/.

### Identification of polymorphic regions among olive cultivars

To identify sequence polymorphisms, the following potentially variant domains were tested: i) regions containing mono-, di-, tetra- and penta-nucleotide microsatellites; ii) regions previously reported as polymorphic among *Olea *subspecies, iii) regions containing high sequence variations among 12 species (see materials and methods for chloroplast sequencing strategy); iv) barcoding regions previously identified for species discrimination that had never been tested in olive cultivars; and v) plastid ESTs derived from massive sequence analyses of fruit cDNAs [55].

Candidate SSRs were selected among those having the highest number of repeats (Table 1 and Figure 2). Although no mono-nucleotide SSRs with repeats shorter than 10 bp were considered, some were indirectly included in the analyses of other regions.

PCR amplifications were performed in a final volume of 25 μl containing 25 ng of template DNA, 2,5 μl of 10 × PCR buffer, 0.5 mM of each dNTP, 1 μM of each primer and 1.5 U/μl of PerfectTaq DNA Polymerase (5-PRIME). The amplifications were run on a thermal cycler Mastercycler Gradient (Eppendorf) using the same conditions as previously indicated for plastid sequencing.

After an initial evaluation by electrophoresis on a 2% agarose gel, amplicons were sequenced in both directions using the ABI Prism BigDye Terminator V.3.1 Ready Reaction Cycle Sequencing Kit and run on an ABI 3130 Genetic Analyzer (Applied Biosystems-Hitachi).

The sequences of each region were aligned to evaluate the presence of SNPs, indels or polymorphic microsatellites among the six cultivars. To use these polymorphisms as chloroplast markers able to distinguish olive cultivars from each other, specific primers localizing within conserved flanking regions were constructed (Additional File 1, Table S1). The resulting fragments ranged in size from 145 to 688 bp and could be amplified at an annealing temperature of 60°C. Some amplicons included from two to five polymorphisms. All 40 polymorphisms can be amplified by a set of 21 primer pairs.

## Authors' contributions

CGNM^1 ^and MDC^2^: contributed to the DNA sequencing of the entire plastome.

MR^1^: conducted all the experiments to establish chloroplast variation at varietal level.

AR^1^: conducted bioinformatic analyses, contributed to the DNA sequencing of the IR and SSC of plastome and revised the manuscript.

LB^1^: conceived the study and wrote the manuscript.

All authors read and approved the final manuscript.

## Author details

^1 ^CNR - Institute of Plant Genetics, 06128 Perugia, Italy

^2 ^University of Cordoba - Dep. of Agronomy, 14071 Cordoba, Spain

## Supplementary Material

Additional file 1 **Table S1 and Table S2**. Supplemental tables in a Word DOC. Click here for file
