# Supplementary material for: Identification of new polymorphic regions and differentiation of cultivated olives (Olea europaea L.) through plastome sequence comparison
Source: BMC Plant Biol. 2010 Sep 24;10:211. doi: 10.1186/1471-2229-10-211 (PMC2956560; doi:10.1186/1471-2229-10-211)
Supplement: Additional file 1 — Table S1 and Table S2. Supplemental tables in a Word DOC. [file 1471-2229-10-211-S1.DOC]

Table S1 Position, amplicon length and primer sequences of the chloroplast olive inter-varietal markers.

| **Start base**^(1)^ | **Length**^(1)^ |  | **Polymorphic sites** | **Primer name** | **Primer sequences** |
| --- | --- | --- | --- | --- | --- |
| 155,838 | 353 bp |  | P1, P2 | OeP1-2_fw | AAAATAGAATTTCTTTCTTCGTCTTTACA |
|  |  |  |  | OeP1-2_rev | TGCATTTTTATTTTCATTTATCTACCC |
| 371 | 207 bp |  | P3, P4 | OeP3-4_fw | TTCGGTAATGTAACGAATAAAAGTCT |
|  |  |  |  | OeP3-4_rev | GCTTCTATCGAAGTTCCATCTACAA |
| 4516 | 296 bp |  | P5 | OeP5_fw | CGAGGTAATCAAACAATTGAGCTA |
|  |  |  |  | OeP5_rev | CGAACAAATATAATTCAGTGAAGTGG |
| 4,787 | 320 bp |  | P6, P7 | OeP6-7_fw | CCACTTCACTGAATTATATTTGTTCG |
|  |  |  |  | OeP6-7_rev | TGTGGTTTTCGTTGGAATTTG |
| 8,912 | 270 bp |  | P8 | OeP8_fw | GGGTCTCCCACAATATCGAA |
|  |  |  |  | OeP8_rev | GGAATCCAAAGATGAAGAGAGAAA |
| 9,414 | 253 bp |  | P9, P10, P11, P12, P13 | OeP9-10-11-12-13_fw | GTCCACTCAGCCATCTCTCC |
|  |  |  |  | OeP9-10-11-12-13_rev | CCCGGCTAGGTACTGACCA |
| 9,768 | 295 bp |  | P14 | OeP14_fw | TCAAGCAACAACAAAAAGAAGAAA |
|  |  |  |  | OeP14_rev | TTGGATCATCAAAGAATCATCAA |
| 12,663 | 311 bp |  | P15 | OeP15_fw | CGGTACCAGTATTTACAATCTTTACTT |
|  |  |  |  | OeP15_rev | GCGATTAATCAAGTCCGACAA |
| 17,365 | 278 bp |  | P16, P17, P18 | OeP16-17-18_fw | ATGGCCAAAATGAACTCCTG |
|  |  |  |  | OeP16-17-18_rev | CCTAAGAGCAGATCCATCTTTTTGA |
| 23,921 | 145 bp |  | P19 | OeP19_fw | CCTTGGAATTCATAAGGGATTTACT |
|  |  |  |  | OeP19_rev | AAGCGCAAATATGTCATGGTTA |
| 32,573 | 351 bp |  | P20, P21 | OeP20-21_fw | TTTTGATTCGTCATCCATATTCA |
|  |  |  |  | OeP20-21_rev | CCCCTGAACTAAATTCTTAGAGAAAA |
| 37,905 | 353 bp |  | P22, P23 | OeP22-23_fw | ACCTATTCGTCCCAGATCCAA |
|  |  |  |  | OeP22-23_rev | ATCGAACCCGCATCTTCTC |
| 43,746 | 610 bp |  | P24, P25, P26 | OeP24-25-26_fw | AGTGTTCGGATCTATTATGACATAGC |
|  |  |  |  | OeP24-25-26_rev | TGAAGCACATAATTGGTTGAAGA |
| 56,876 | 338 bp |  | P27, P28 | OeP27-28_fw | CGAATTCGAACCTGAACTCTATTT |
|  |  |  |  | OeP27-28_rev | TGTTTGATTATTAGACCATGGTATTTG |
| 65,533 | 252 bp |  | P29 | OeP29_fw | TCCAGTTCGTAAAAAGAACCAAA |
|  |  |  |  | OeP29_rev | GGCAATTGAAATCTGGCAATA |
| 66,263 | 157 bp |  | P30 | OeP30_fw | CAGGTGTTCTGAATCAATCAATG |
|  |  |  |  | OeP30_rev | TTGTCTGATTCGAGGGGAAA |
| 83,035 | 375 bp |  | P31, P32, P33 | OeP31-32-33_fw | CTCAGCAATAGTGTCCCTACCC |
|  |  |  |  | OeP31-32-33_rev | TGATAATACGATATGATGACAATGCT |
| 101,043 | 341 bp |  | P34 | OeP34_fw | CAAGAGATCTTTCTCGATCAATCC |
|  |  |  |  | OeP34_rev | TCGATTCATACAGAAGAAAAGGTTC |
| 114,320 | 648 bp |  | P35, P36 | OeP35-36_fw | TGGTCCAAGACCATACATATTGA |
|  |  |  |  | OeP35-36_rev | TTCAAAATGAATCCGTAGTTTCCT |
| 115,297 | 371 bp |  | P37, P38 | OeP37-38_fw | TCGCTCCGCTCTTAGAATATG |
|  |  |  |  | OeP37-38_rev | TATGGGGGTGTATATAGGAATTCAG |
| 127,690 | 688 bp |  | P39, P40 | OeP39-40_fw | TTGACTTGTTTCATTATTGTAAATGGA |
|  |  |  |  | OeP39-340_rev | CCCGAAAACGACCCTCTT |

^(1)^ The start base and the length of the amplified region refers to the cv. Frantoio sequence.

**Table S2 Primers and amplicons used to amplify all overlapping regions of the entire *Olea europaea*** chloroplast genome.

| **Position**^(1)^ | **Primer** | **Sequence** | **Fragment size (bp)**^(1)^ | **Region** |
| --- | --- | --- | --- | --- |
| 83,437 | LSC-IRb-f1 | CAGGTACGAACAATTACAGCTCTGACTAC | 4,721 | LSC |
| 88,158 | LSC-IRab-f1 | TATACAAAACTTCTACCCCGAGCACACG |  | IR |
| 87,987 | OeIRb-1f | TTACGGTATAGACGCTTATGACCTCC | 2,955 | IR |
| 90,942 | OeIRb-1b | TGATTGACTGCCYCCATTATGTTGTT |  | IR |
| 90,655 | OeIRb-2f | TCKATTCACTCTATCAATAASCGAGC | 3,475 | IR |
| 94,130 | OeIRb-2b | GAATATGRGTCGAWGCAATAACAAGA |  | IR |
| 93,685 | OeIRb-3f | CTATTTGKTCAAATAYCTAGCGRCAA | 3,866 | IR |
| 97,551 | OeIRb-3b | CTATTACTCAAACAAGCATGAAACGT |  | IR |
| 97,324 | OeIRb-4f | TYGTGTATAATCCTGCATAATCTCGA | 3,506 | IR |
| 100,830 | OeIRb-4b | CGTATGAGRTGAAAATCTCAYGTACG |  | IR |
| 100,533 | OeIRb-5f | CGCCCTTGTTGACGATCCTTTACTCC | 3,442 | IR |
| 103,975 | OeIRb-5b | ACCGCCGTATGGCTGACCGGCGATTA |  | IR |
| 103,703 | OeIRb-6f | CAACGAGCGCAACCCTCGTGYTTAGT | 3,133 | IR |
| 106,836 | OeIRb-6b | CCACAACCCCGTTTTCRCGGTTTAGG |  | IR |
| 106,564 | OeIRb-7f | AACRARGAAAGGCTTACGGTGGATAC | 3,032 | IR |
| 109,596 | OeIRb-7b | ATTGATCRGGTCATGTAGGAACAAGG |  | IR |
| 109,328 | OeIRb-8f | AAGCATCTAAGTARTAAGCCCACCCC | 2,343 | IR |
| 111,671 | OeIRb-8b | AAATGAGATTATTCAGGAATACRCATTG |  | IR |
| 111,497 | OeIRb-9f | GCGCCTCTGCATCTAGCATTGGGTA | 2,028 | IR |
| 113,525 | OeSSC1b | GCTTTRTTTCATTTGATTACTCATGC |  | SSC |
| 113,312 | OeSSC-2f | ARGCAAGRGGBGGAATACCACAAAGA | 4,463 | SSC |
| 117,775 | OeSSC-2b | TTWTCVATGGCBTCYCTTGCATTRCC |  | SSC |
| 117,588 | OeSSC-3f | CCATAGAACATYTGGCGTRACATAGA | 2,980 | SSC |
| 120,568 | OeSSC-3b | GGGTTTRYCTGRACCAATACAYGATT |  | SSC |
| 120,327 | OeSSC-4f | GAACATCACAGCAAAWADRATTAAAA | 3,414 | SSC |
| 123,741 | OeSSC-4b | AGAATGAAATTGGCYGATATTATGAC |  | SSC |
| 123,475 | OeSSC-5f | GGHCCRATACGTTGTTGTATYCCTGC | 1,644 | SSC |
| 125,119 | OeSSC5bbis | AGACTTACTTCACATTTGRAATTGCACAA |  | SSC |
| 124,860 | OeSSC51f | TACCGAGTCATTCTTCCATGAATTGC | 614 | SSC |
| 125,474 | OeSSC51b | AAATGAATCAACAGAACCCTCTTC |  | SSC |
| 125,067 | OeSSC-6f | CCTCTYTGAGATRAATAGTCTKTTYTGTGC | 3,583 | SSC |
| 128,650 | OeSSC-6bbis | TGAATGGTATGAGGATTTAGAGGAATGGAA |  | SSC |
| 128,513 | OeSSC-7f | AAAGGAWATARDATYTTTATYTGAATACC | 2,315 | SSC |
| 130,828 | OeIRa-7b | ATTATTCAACCATTTCATTTTACCAAG |  | IRa |
| 154,346 | LSC-IRab-f1 | TATACAAAACTTCTACCCCGAGCACACG | 2,188 | IRa |
| 644 | LSC-IRa-r1 | AACCGTGCTAACCTTGGTATGGAAG |  | LSC |
| 155,638 | OeIRa-LSC-f2 | TGGCTAGGTAAGCGTCCTGT | 2,187 | IRa |
| 1,936 | OeLSC-IRa-b2 | GCTTGGGGAGGGGTTTTTCC |  | LSC |
| 5,985 | OeLSC-f3 | CTTTCAAAATGGCAGCAACA | 3,576 | LSC |
| 9,561 | OeLSC-b3 | CTCGTTCGAGCCCTTCCTTT |  | LSC |
| 8,489 | OeLSC-f4 | TCCAAGAACAAAATGTCTGTTATGC | 6,504 | LSC |
| 14,993 | OeLSC-b4 | TTCCATCTCGTTGTGACTGTG |  | LSC |
| 14,249 | OeLSC-f5 | CTACGAACGGGAAAGGATGA | 5,001 | LSC |
| 19,250 | OeLSC-b5 | CCGTGCTTCCTTTGTTGAAA |  | LSC |
| 17,941 | OeLSC-f6 | TCGCTTCAGATATGAAACTTTGAG | 7,105 | LSC |
| 25,046 | OeLSC-b6 | GGGCTCTAGAAGGATTTGGTG |  | LSC |
| 22,977 | OeLSC-f7 | ACCGTCCCTCATTGGTTGTC | 5,665 | LSC |
| 28,642 | OeLSC-b7 | CCGAATAGACCGGCAAAAA |  | LSC |
| 26,921 | OeLSC-f8 | TCATAAGTGGTTGTTAATGGAGTTGA | 3,903 | LSC |
| 30,824 | OeLSC-b8 | TTGAAAAACGATATCGGCAAA |  | LSC |
| 31,986 | OeLSC-f9 | GCCCCCTGACATCCTTTCAT | 1,280 | LSC |
| 33,266 | OeLSC-b9 | GCCTTACCATGGCGTTACTC |  | LSC |
| 36,324 | OeLSC-f10 | CGGAGGACATGTATGGTTAGG | 6,650 | LSC |
| 42,974 | OeLSC-b10 | CGGGGCTACTAGGACTTGGG |  | LSC |
| 40,618 | OeLSC-f11 | AAAAGTGGGCCTAACCCTTG | 5,585 | LSC |
| 46,203 | OeLSC-b11 | ATTACGAATAATTCCGACAACTTCA |  | LSC |
| 44,466 | OeLSC-f12 | GGTCGGAATAGGTAGGTTAATTCCT | 5,045 | LSC |
| 49,511 | OeLSC-b12 | TCGATTCTTTCTTCAATGTGGA |  | LSC |
| 51,196 | OeLSC-f13 | TCAAAGCCCAAAGATCTATGAATTA | 3,621 | LSC |
| 54,817 | OeLSC-b13 | TTGATCCACAAGAAGCTCAGA |  | LSC |
| 52,455 | OeLSC-f14 | AAAACATAGACGCACTCCTATGAAC | 6,317 | LSC |
| 58,772 | OeLSC-b14 | TCCATTGCTGCAAACTCAAA |  | LSC |
| 55,695 | OeLSC-f15 | ATTCTACCCAATAAGGCCGATACT | 5,771 | LSC |
| 61,466 | OeLSC-b15 | CCAAGGAATGTAGAACTAAATAAATGG |  | LSC |
| 60,327 | OeLSC-f16 | TTGGATTTCATTCGGAGGAG | 5,631 | LSC |
| 65,958 | OeLSC-b16 | AGGAATTTTTGACGAACAGGA |  | LSC |
| 65,821 | OeLSC-f17 | CATAAGTAAGCGGGGAGCAA | 7,907 | LSC |
| 73,728 | OeLSC-b17 | TTTTAGGCCAAGAAGTTGATAGC |  | LSC |
| 72,034 | OeLSC-f18 | CTCTTAGTCCGTTCTTGACAGAGTG | 2,269 | LSC |
| 74,303 | OeLSC-b18 | TGTATCATAAATGATTCCTGTTTGG |  | LSC |
| 76,781 | OeLSC-f19 | TTGTAAACCACGATCGAATCTATG | 5,932 | LSC |
| 82,713 | OeLSC-b19 | ACAGACCGAGAGGCTCGACT |  | LSC |
| 81,109 | OeLSC-f20 | TCAACGATTTCTACATAAGGTGGT | 5,304 | LSC |
| 86,413 | OeIRb-LSC-b2 | GGTAGGACACAAATTGGGAGA |  | IRb |

1. The position and the fragment size of the amplified region refers to the cv. Frantoio sequence.
